# Impaired leukocyte influx in cervix of postterm women not responding to prostaglandin priming

**DOI:** 10.1186/1477-7827-6-36

**Published:** 2008-09-02

**Authors:** Lena Sahlin, Ylva Stjernholm-Vladic, Nathalie Roos, Britt Masironi, Gunvor Ekman-Ordeberg

**Affiliations:** 1 Division for Reproductive Endocrinology, Q2:08, Karolinska University Hospital – Solna, Karolinska Institutet, Stockholm, Sweden; 2 Division of Obstetrics and Gynecology, H2:01, Department of Woman and Child Health, Karolinska University Hospital – Solna, Karolinska Institutet, Stockholm, Sweden

## Abstract

**Background:**

Prolonged pregnancies are associated with increased rate of maternal and fetal complications. Post term women could be divided into at least two subgroups, one where parturition is possible to induce by prostaglandins and one where it is not. Our aim was to study parameters in cervical biopsies in women with spontaneous delivery at term (controls) and compare to those that are successfully induced post term (responders), and those that are not induced (non-responders), by local prostaglandin treatment.

**Methods:**

Stromal parameters examined in this study were the accumulation of leukocytes (CD45, CD68), mRNAs and/or proteins for the extracellular matrix degrading enzymes (matrix metalloproteinase (MMP)-2, MMP-8 and MMP-9), their inhibitors (tissue inhibitor of MMP (TIMP)-1 and TIMP-2), interleukin-8 (IL-8), the platelet activating factor-receptor (PAF-R), syndecan-1 and estrogen binding receptors (estrogen receptor (ER)α, ERβ and G-coupled protein receptor (GPR) 30) as well as the proliferation marker Ki-67.

**Results:**

The influx of leukocytes as assessed by CD45 was strongest in the responders, thereafter in the controls and significantly lower in the non-responders. IL-8, PAF-R and MMP-9, all predominantly expressed in leukocytes, showed significantly reduced immunostaining in the group of non-responders, while ERα and GPR30 were more abundant in the non-responders, as compared to the controls.

**Conclusion:**

The impaired leukocyte influx, as reflected by the reduced number of CD45 positive cells as well as decreased immunostaining of IL-8, PAF-R and MMP-9 in the non-responders, could be one explanation of the failed ripening of the cervix in post term women. If the decreased leukocyte influx is a primary explanation to absent ripening or secondary, as a result of other factors, is yet to be established.

## Background

The human uterine cervix is an extracellular matrix (ECM) organ. The cervical ripening at term is clinically recognized as softening and dilation. This process corresponds to a remodelling of the cervical ECM with a changed proteoglycan composition and an increased collagen turnover resulting in differently organized collagen fibrils [1,2]. This remodelling of the cervix is necessary for a normal onset and progress of parturition.

The ripening process is characterized by inflammatory events, such as extravasation of neutrophils and macrophages [3-5] and an increased cervical level of pro-inflammatory cytokines such as interleukin (IL)-8 [4,6]. Post-term pregnancies, gestational length of >42 weeks, are associated with increased rates of maternal and fetal complications [7,8]. Among mothers delivering post term there is a significant risk in subsequent post term births, indicating that there are factors, genetic or other, that influence pregnancy length [9]. It is common practice worldwide to induce parturition when gestational age increases beyond 41 weeks, since the risk of complications is increasing after week 41 [10,11]. In Sweden there is a consensus to induce labour after 42 weeks of gestation. Most women respond to cervical prostaglandin application and labour is induced, but there is a group of women where no progress is achieved [12].

In previous studies we have shown that expression of estrogen receptor (ER)α and ERβ are varying in cervix biopsies obtained from women that are non pregnant, term pregnant or immediately postpartum [3,13]. Recently several articles regarding a specific estrogen receptor in the cell membrane, the G-protein coupled receptor 30 (GPR30) have been published [14-16]. GPR30 has been described in endometrial and ovarian cancer cells, but to our knowledge nothing is known from in vivo expression in the human cervix.

Platelet-activating factor (PAF) is a lipid pro-inflammatory mediator, involved in several reproductive processes, i.e. parturition [17]. PAF is a phospholipid synthesized by leukocytes, blood platelets and vascular endothelial cells [18]. PAF-R is a G-protein coupled membrane receptor with an estrogen responsive element within its promoter region, enabling regulation by estrogens [19]. We have found that immunostaining of PAF-R was higher in cervical stroma in term pregnant women than immediately after delivery [20]. The activation of the PAF-R is associated with cytoskeletal remodelling and expression of pro-inflammatory modulators, such as COX-2, IL-6 and IL-8 [21].

In a previous study we identified cervical stromal fibroblasts and smooth muscle cells as main sources of matrix metalloproteinase (MMP)-2, whereas the MMP-9 protein was observed exclusively in invading leukocytes [22]. Both MMPs were shown to be increased in the end of pregnancy as compared to cervical biopsies from non-pregnant women [22]. These data indicate the involvement of MMP-2 and MMP-9 in the cervical ripening process. The MMP family consists of more than 20 members with broad substrate specificities [23]. Main substrates for collagenases (MMP-1, -8, and -13) are fibrillar and non-fibrillar collagens. Primary targets of gelatinases (MMP-2 and -9) are denatured collagen IV, elastin, proteoglycans and fibronectin [24]. Syndecan-1 (S-1) is a cell surface proteoglycan that binds cells to the extracellular matrix and acts as a regulator of chemokine function [25]. In rat uterus, using microarray technology, we found that S-1 was clearly estrogen dependent [26]. Ki-67 is a marker for proliferation, and estrogens are known to induce cellular proliferation [27]. Thus, detection of S-1 and Ki-67 could indicate level of estrogen stimulation.

The aim of this study was to determine the parameters described above, in the cervix at term after spontaneous vaginal delivery in comparison to that of women with unripe cervices post term, both those responding to prostaglandin treatment and those who did not respond, to find out possible differences.

## Methods

All women were nulliparous healthy, non-smoking, had uncomplicated pregnancies and were without medication. The gestational length was estimated according to ultrasound dating in the second trimester of gestation.

The post term groups consisted of women with an unripe cervix defined as a Bishop score ≤ 5 points. Prostaglandin-E_2 _in viscous gel (Minprostin^® ^Pharmacia, Sweden) was administered locally to induce cervical priming. Oxytocin infusion (Syntocinon^® ^10 U/glucose 5.5% 500 mL) for augmentation of labour was administered to every woman in the responder group 5 h after the latest prostaglandin application, to a five out of ten women in the non-responder group 5 h after the latest prostaglandin application and to all woman in the control group during the active phase of labour, if failure to progress after 2 h according to the clinical guidelines [28].

The group that responded to Miniprostin treatment (responders = R, n = 13), i.e. who underwent vaginal parturition after successful cervical priming and labour induction (biopsy obtained immediately post partum) had a mean age (± SD) of 29.4 years ± 5.8, a median gestational length of 42+4 weeks (range 42+1 to 42+6), and a median Bishop score of 3 points (range 0–4) before treatment. This group received a median of total prostaglandin treatment of 1.0 mg (range 1.0–2.0).

The group of women who did not respond (non-responders = NR, n = 10) to Miniprostin priming, i.e. experienced a failed induction (biopsy taken after caesarean section), had a mean age of 30.6 years ± 1.9, a median gestational length of 42+4 weeks (range 42+3 to 42+6) and a mean Bishop score of 2 points (range 0–4) before treatment. These women received a median of 4.0 (range 0.5–7.0) mg of prostaglandin treatment.

As controls we collected biopsies immediately after delivery from women who had a spontaneous onset of labour and underwent vaginal delivery at normal gestational length (controls = C, n = 18). These women had a mean age of 29.3 years ± 4.9 and a median gestational age of 40+1 weeks (range 37+0 to 41+1).

Cervical biopsies (150–300 mg) were taken transvaginally at the 12 o'clock position at partus or immediately after emergency caesareans, by one physician (YSV). The study was approved by the Local Ethics Committee (Dnr 99–099), and informed consent was obtained from all women before biopsies were collected. The biopsies were when possible divided into 2 pieces, one immediately frozen and the other was immersion-fixed in 4 % formaldehyde at 4°C overnight, stored at 4°C in 70% ethanol and thereafter embedded in paraffin. When the biopsy was too small to be divided, only a fixed sample was prepared, therefore RNA preparations are not available from all samples.

IL-8 immunostaining was also performed in samples from a previously published study, for details on the non-pregnant (NP), term pregnant (TP) and post partum (PP) groups, see Wang et al. [13]. The PP group is similar to the control group of the post term study, i.e. biopsies are obtained immediately after spontaneous vaginal delivery at term.

### RNA preparation and reverse transcription

Total RNA from frozen cervical tissue samples was purified with the RNeasy^® ^kit (Qiagen GmbH, Hilden, Germany) according to a procedure recommended by manufacturer for RNA isolation from fibrous tissues. Two μg of total RNA from each sample was reverse transcribed at 37°C for 60 min in a final volume of 30 μl with a reaction mixture (Qiagen GmbH, Hilden, Germany) containing 1 × RT buffer, dNTP mix (0.5 mM each dNTP), 600 ng random primers (Invitrogen, Paisley, UK), 10 units RNase inhibitor (Superase-In, Ambion, Austin, TX), and 4 U of Omniscript™ reverse transcriptase (Qiagen).

### Real time PCR analysis

The oligonucleotide primers for ERα, ERβ, GPR30, MMP-2, MMP-8, MMP-9, TIMP-1, TIMP-2, S-1, PAF-R and Cyclophilin A are presented in Table 1, as well as their predicted sizes. Real time PCR was performed in a DNA Engine Opticon™ 2 System (MJ Research, Waltham, MA). For PCR, the cDNAs corresponding to 40–100 ng (see Table 1) RNA were added to 10 μl of Quantitect™ SYBR^® ^Green PCR mix (Qiagen) containing HotStarTaq DNA polymerase, PCR buffer, dNTP mixture and 0.3 μM of each oligonucleotide primer in a final volume of 20 μl. The reactions were performed in opaque white 0.2 ml low-profile strip tubes sealed with optical flat caps (TLS-0851, TCS-0803, MJ Research, Waltham, MA). After initial incubation for 15 min at 95°C, the samples were subjected to 40–44 cycles of 10s at 94°C, 15–20s at 55–58°C (see Table 1) and 20s at 72°C with a final extension step at 72°C for 5 min. All reactions were performed twice. The purity of PCR products was confirmed by a melting curve analysis in all experiments (data not shown). The oligonucleotide primers are listed in Table 1. All primers were designed to span an intron/exon boundary or to flank an intron, thus, amplification of contaminating DNA was eliminated. Each PCR assay included a negative control containing a RNA sample without reverse transcription. The primers were based on the sequences of the human genes. The primer pairs (Table 1) were designed with Primer3 software [29].

**Table 1:** Oligonucleotide primers used for real-time PCR, template amount and their annealing temperatures.

**Gene**	**Accession No. or Reference**	**Primer** F = forward; R = reverse	**Position**	**cDNA**	**Annealing step**
MMP-2	BC002576	F: gtatttgatggcatcgctca	bp 1695-1714	40 ng	56°C/20s
		R: cattccctgcaaagaacaca	bp 1891-1871		
			product bp 198		
MMP-8	NM_002424	F: ctttcagggaaaccagcaac	bp 790-809	80 ng	55°C/20s
		R: gcttggtccagtaggttgga	bp 893-874		
			product bp 104		
MMP-9	BC006093	F: cgctaccacctcgaactttg	bp 1124-1143	40 ng	56°C/20s
		R: gccattcacgtcgtccttat	bp 1319-1300		
			product bp 198		
TIMP-1	NM_003254	F: tgacatccggttcgtctaca	bp 299-318	40 ng	56°C/20s
		R: tgcagttttccagcaatgag	bp 400-381		
			product bp 102		
TIMP-2	PMID 15231657 [50]	F: gagcctgaaccacaggtacca	bp 728-748	40 ng	58°C/15s
		R: tctgtgacccagtccatcca	bp 841-822		
			product bp 114		
PAF-R	NM_000952	F: cagagacacacggtcactgc	bp 44-63	40 ng	56°C/15s
		R: catgtgggaggagtcatgtg	bp 154-135		
			product bp 111		
S-1	NM_002997	F: ggagcaggacttcacctttg	bp 869-888	50 ng	59°C/30s
		R: cccagcacctctttcctgt	bp 1009-991		
			product bp 141		
ERα	NM_000125	F: cttgctcttggacaggaacc	bp 1581-1600	80 ng	56°C/20s
		R: tcctctccctgcagattcat	bp 1691-1672		
			product bp 111		
ERβ	NM_001437	F: tgcggaacctcaaaagagtc	bp 715-734	80 ng	56°C/20s
		R: catccctctttgaacctgga	bp 854-835		
			product bp 140		
GPR30	NM_001505	F: agactgtgaaatccgcaacc	bp 364-383	100 ng	57°C/15s
		R: aagtgagcctggcatttgt	bp 663-644		
			product bp 300		
Cyclophilin A	XM_004890	F: gtggtgtttggcaaagtgaa	bp 401-420	40 ng	56°C/20s
		R: tcgagttgtccacagtcagc	bp 516-497		
			product bp 116		

### Quantification of mRNA

To standardize the quantification method, cyclophilin A was selected out of several tested housekeeping genes as an invariable internal control (p = 0.482). The PCR amplification rate and the cycle threshold (Ct) values were related to a standard curve using Opticon Monitor 3.0 software (MJ Research, Waltham, MA). The values of relative expression of genes of interest were normalized against the cyclophilin A product.

### Immunohistochemical analysis

Immunostaining for the determination of ERα, ERβ, GPR30, MMP-9, cd45, cd68, S-1, Ki-67, IL-8 and PAF-R utilizing the avidin-biotin peroxidase complex (ABC) procedure was performed [30]. The 5 μm paraffin sections prepared from cervical biopsies were first dewaxed in Bioclear (Bio-Optica, Milan, Italy), rehydrated and washed with phosphate-buffered saline (PBS; pH 7.4)) (ERβ and PAF-R in Tris-buffered saline (TBS)). Thereafter the sections were subjected to microwave antigen retrieval in 0.01 M sodium citrate buffer (pH 6.0) for 10 min and then allowed to cool for 20 min. Subsequently, endogenous peroxidase activity was quenched by immersion in 3% hydrogen peroxide (Merck) in methanol for 10 min at room temperature; followed by blocking non-specific binding of the primary antibody by incubation as shown in Table 2, at room temperature (RT). The sections were then incubated with the primary antibodies (see Table 2). For the negative controls the primary antibody was replaced by mouse IgG (or in the case of GPR30 rabbit IgG) at a corresponding concentration to the antibody it replaced.

**Table 2:** Antibodies used in the study.

**Protein**	**Order number and company**	**Type**	**Dilution**	**Incub. time**	**Blocking**
CD45RB	M0833 (*B cells, T cells subsets, monocytes, macrophages and some granulocytes*) DAKO A/S.	Mc mouse anti-human	1:200	+4°C o/n	D
CD68	M0876 (*macrophages*) DAKO A/S, Glostrup, Denmark	Mc mouse anti-human	1:200	+4°C o/n	D
MMP-9	MS-817-P0, NeoMarkers Inc	Mc mouse anti-human	1:400	RT 60 min	D
IL-8	sc-73321, Santa Cruz Biotechnology, Inc	Mc mouse anti-human	1:200	+4°C o/n	A
PAF-R	160600, Cayman chemical,	Mc mouse anti-human	1:350	+4°C o/n	E
S-1 (CD138)	M7228, DakoCytomation,	Mc mouse anti human	1:50	RT 30 min	A
ERα	2nd Gen 08-1149, Zymed Laboratories Inc	Mc mouse anti-human	1:5	+4°C o/n	A
ERβ	Serotec Ltd, MCA1974	Mc mouse anti-human	1:20	+4°C o/n	B
GPR30	LS-A4272, LifeSpan BioSciences	Pc rabbit anti-human	1:300	RT 60 min	C
Ki-67	NCL-Ki67-MM1, Novocastra Laboratories Ltd	Mc mouse anti human	1:200	+4°C o/n	A

Mc = monoclonal; Pc = polyclonal, A = 1.5% normal horse serum (NHS) in PBS for 30 min, B = 1.5% normal horse serum (NHS) in TBS containing 5% bovine serum albumin (BSA) for 45 min, C = 1.5% normal goat serum (NGS) in PBS containing 5% bovine serum albumin (BSA) for 30 min, D = 1.5% normal horse serum (NHS) in PBS for 45 min, E = 1.5% normal horse serum (NHS) in TBS for 45 min.

NHS = normal horse serum (Vector Laboratories, Burlingame, CA).

NGS = normal goat serum (DakoCytomation, Glostrup, Denmark).

The secondary biotinylated antibody (Table 3) was incubated as shown in Table 3, followed by incubation with an avidin-biotin horseradish peroxidase complex (Vectastain Elite, Cat# PK-6100) for 30 min at RT. The site of the bound enzyme was visualized by the application of 3,3'-diaminobenzidine (DAB kit, DAKO, CA), a chromogen which produces a brown, insoluble precipitate when incubated with enzyme. The sections were counterstained with haematoxylin and dehydrated before they were mounted with Pertex (Histolab, Gothenburg).

**Table 3:** Secondary anti-bodies used in the study, their dilution, buffers and incubation time.

**Protein**	**2nd Ab**	**Dilution**	**Buffer**	**Incubation**
CD45RB	biotinylated horse anti-mouse	1:200	1.5% NHS in PBS	60 min RT
CD68	biotinylated horse anti-mouse	1:200	1.5% NHS in PBS	60 min RT
MMP-9	biotinylated horse anti-mouse	1:200	1.5% NHS in PBS	60 min RT
IL-8	biotinylated horse anti-mouse	1:200	1.5% NHS in PBS	30 minRT
PAF-R	biotinylated horse anti-mouse	1:200	1.5% NHS in TBS	30 min RT
S-1 (CD138)	biotinylated horse anti-mouse	1:200	1.5% NHS in PBS	30 min RT
ERα	biotinylated horse anti-mouse	1:200	1.5% NHS in PBS	30 minRT
ERβ	biotinylated horse anti-mouse	1:200	1.5% NHS in TBS + 5% BSA	60 min RT
GPR30	biotinylated goat anti-rabbit	1:200	1.5% NGS in PBS + 5% BSA	30 min RT
Ki67	biotinylated horse anti-mouse	1:200	1.5% NHS in PBS	30 min RT

biotinylated horse anti-mouse antibody (Vector, Cat# BA-2000).

biotinylated goat anti-rabbit antibody (Vector, Cat# BA-1000).

To obtain negative controls for the immunohistochemistry assays, the mouse monoclonal antibodies were replaced by a normal mouse IgG of the same concentration as the primary antibody. The polyclonal GPR30 antibody was replaced by normal goat IgG of equal concentration.

### Image analysis

A Leica microscope and Sony video camera (Park Ridge, NJ, USA) connected to a computer with an image analysis system (Leica Imaging System Ltd, Cambridge, UK) was used to assess quantitative values from immunohistochemistry. The quantification of immunostaining was performed as described previously [30]. Thus, quantification of immunostaining was performed on the digitized images of systematic randomly selected fields of cervical stroma, from which non-stromal elements (e.g. epithelium and glands) were interactively removed. Ten to 12 fields of stromal cells were measured separately in each tissue section. By using color discrimination software, the total area of positively stained cells (brown reaction product) was measured, and expressed as a ratio of the total area of cell nuclei (brown reaction product + blue haematoxylin).

### Manual scoring

Two observers blinded to the identity of the slides (LS, BM) performed all the assessments. The staining was evaluated semi-quantitatively using a grading system. The staining intensity and amount of positive cells were graded on a scale of (0) no staining, (1) faint/few positive cells, (2) moderate/20–50% cells positive and (3) strong staining/majority of cells positive.

### Statistical analysis

Statistical calculation was performed by a parametrical method (one way ANOVA followed by Scheffés test to evaluate significance) for patient age. For all other parameters statistical calculations were performed by the non-parametric method ANOVA on ranks (Kruskal-Wallis test) and significances were evaluated by Dunn's test. Values are considered significantly different when *P *< 0.05.

## Results

There was no difference between the study groups regarding age, and no difference between the R and NR groups in the Bishop score before treatment. The gestational age of the control group (40+1) was significantly shorter than that of the R and NR groups (42+4 in both). The NR group received, as compared to the R group, significantly more prostaglandin (4 to 1 mg in total) and had more times of application (3 to 1.5 times) before delivery.

Due to the very small biopsies we could collect there were just four samples available for the NR group in the PCR assays, why the power of those calculations is less than the 0.80 that is desired. Therefore we are less likely to detect a difference when one actually exists. We show the results for the mRNA determinations, even if there are no significant differences, to enable for the viewer to judge and to allow comparison with immunostaining results. In addition, the mRNA levels are determined in a RNA preparation from a homogenate of the cervical biopsy, why potential differences between different cell types could even out each other.

### Leukocytes

**Figure 1:**
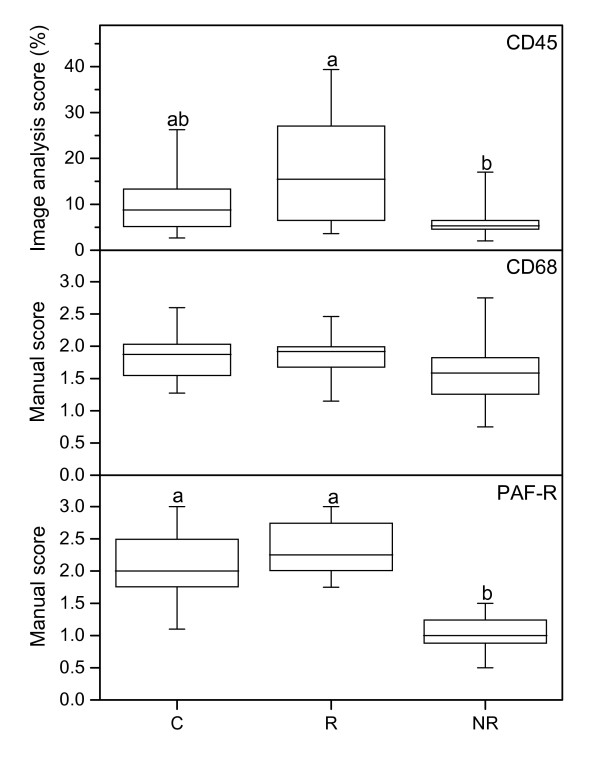
**Immunostaining results of CD45, CD68 and PAF-R**. CD45 (top), CD68 (middle) and PAF-R (bottom) immunostaining results in stroma, as assessed by image analysis (top) and manual scoring (middle, bottom) in cervical samples from controls (C) (n = 18), responders (R) (n = 13) and non-responders (NR) (n = 9). The "box-and-whisker plot" represents the median value with 50% of all data falling within the box. The whiskers extend to the 5th and 95th percentiles. Boxes with different letter designations are significantly different, p < 0.05.

**Figure 2:**
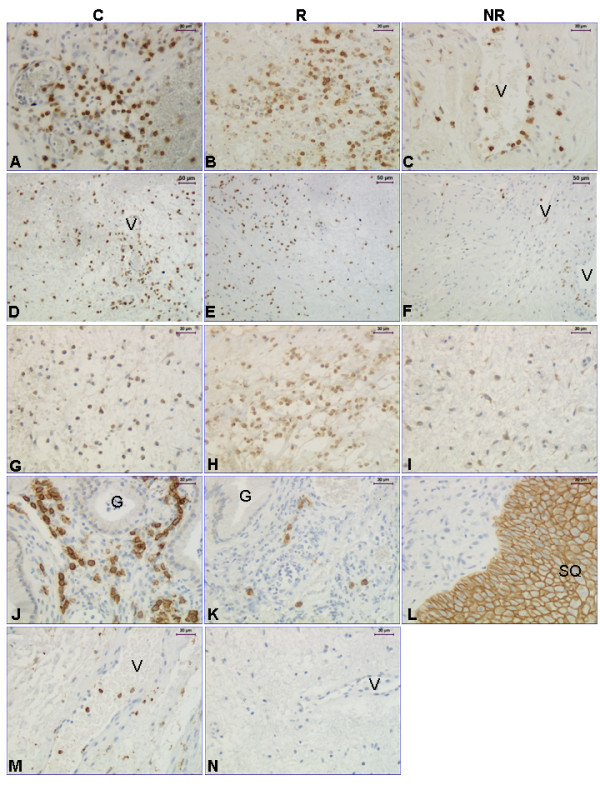
**Representative images of CD45, MMP-9, PAF-R, syndecan-1 and CD68 immunostaining**. Representative images of the immunostaining results for CD45 (**A-C**), MMP-9 (**D-F**), PAF-R (**G-I**), Syndecan-1 (**J-L**) and CD68 (**M**). A negative control is shown for monoclonal antibodies (**N**) where the primary antibody (in this example CD45) was replaced by an equal amount of mouse IgG. Abbreviations: G: gland; V: vessel and SQ: squamous epithelium. The magnification bars represent 30 μm in all images but D-F, where it is 50 μm.

Immunostaining of CD45 showed that there are less leukocytes in the NR group than in the responders (Figure 1, upper panel; Figure 2A–C), indicating that prostaglandin treatment, when successful, induces leukocyte influx. CD68 immunostaining (Figure 2M) showed no significant difference between groups (Figure 1, middle panel).

### MMPs and TIMPs

**Figure 3:**
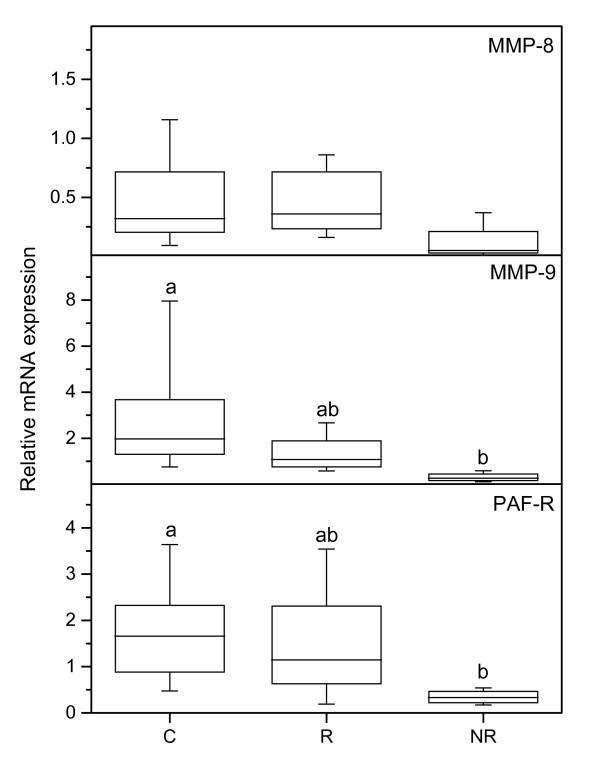
**PCR results for MMP-8, MMP-9 and PAF-R**. Representative real-time PCR experiments for expression of MMP-8 (upper), MMP-9 (middle) and PAF-R (bottom) mRNAs in human cervix from the C (n = 11), R (n = 10) and NR (n = 4) groups respectively. The values of relative expression of target genes were normalized against cyclophilin A and displayed in arbitrary units. The "box-and-whisker plot" represents the median value with 50% of all data falling within the box. The whiskers extend to the 5th and 95th percentiles. Boxes with different letter designations are significantly different, p < 0.05.

No significant differences in MMP-2 mRNA levels between the study groups were found (p= 0.57). There was a tendency to a decreased level of MMP-8 mRNA in the NR group as compared to the C and R groups (p = 0.07) (Figure 3, upper panel). The MMP-9 mRNA level was lower in the NR group as compared to the controls (Figure 3, middle panel). Immunostaining with the MMP-9 antibody showed that the leukocytes stained positive and scoring was not performed (Figure 2D–F).

There were no significant differences in the mRNA levels of TIMP-1 and TIMP-2 between groups (p = 0.103 and p = 0.677, respectively).

### PAF-R

The PAF-R mRNA level was lower in the NR group as compared with the controls (Figure 3, bottom panel). There is also less cells positively stained for PAF-R in the NR group, as compared to both the R and C groups (Figure 1, bottom panel; Figure 2G–I), when scoring the stroma which to a large extent contained positively stained leukocytes.

### IL-8

**Figure 4:**
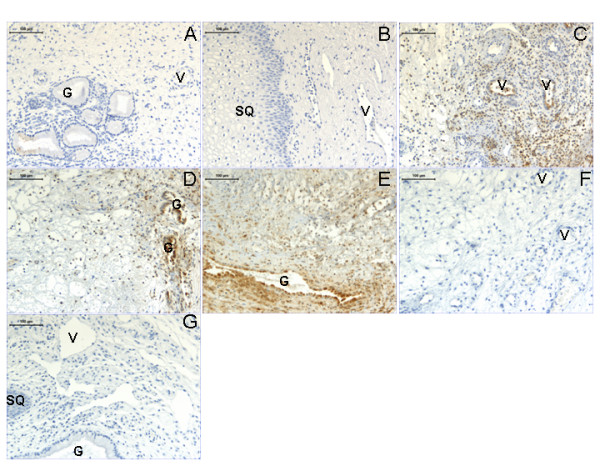
**Representative images of IL-8 immunostaining**. Representative images of IL-8 immunostaining of cervical biopsies from women non-pregnant (**A**), term pregnant (**B**), post partum (**C**). Second row shows images from women delivered spontaneously at term (**D**), post term women responding to prostaglandin priming (R group) delivering vaginally (**E**) and post term women who did not respond to prostaglandin treatment (NR group) and was delivered by caesarean section (**F**). A negative control where the primary antibody was replaced by an equal amount of mouse IgG, is shown in (**G**). Abbreviations: V: vessel; G: gland and SQ: squamous epithelium. Magnification bars represent 100 μm in all images.

Immunostaining of IL-8 showed that there are more leukocytes staining positive in the cervix from women immediately post partum (PP), than in term pregnancy (TP) or non-pregnant (NP) (Figure 4A–C; Table 4). In addition, the glandular epithelium also stained more positive in the PP as compared to the NP group (Table 4). In the non-responders of the present study, there were less IL-8 immunopositive leukocytes as compared to both the responders (R) and the controls (Figure 4D–F; Table 4). A negative control, where the primary antibody was replaced by a equal amount of mouse IgG, is shown in Figure 4G.

**Table 4:** Scoring results of IL-8 immunostaining.

**IL-8**	**Leukocytes** (median and range)	**Glandular epithelium** (median and range)
NP (n = 6) resp (n = 4)	0 (0–1)	0 (0–1)
TP (n = 8) resp (n = 5)	0 (0–1)	1 (0–1)
PP (n = 10) resp (n = 10)	2 (1–2) §	2 (1–3) *

C (n = 18) resp (n = 9)	2 (1–3)	2 (1–3)
R (n = 13) resp (n = 2)	2 (1–3)	
NR (n = 10) resp (n = 1)	0 (0–3) #	

Glandular epithelium (GE) was not present in all samples why the n is less there. In the post term study it was not possible to do statistical calculations of IL-8 immunostaining in GE.

§ = significantly different to NP and TP groups.

* = significantly different to the NP group.

# = significantly different from the C and R groups.

### Syndecan-1

The syndecan-1 (S-1) mRNA level did not differ significantly between the groups. Over all stromal S-1 immunostaining did not differ between the groups, but when we scored stroma close to glands and stroma close to squamous epithelium separately, there were significantly more S-1 immunostaining in the stroma close to glands in the C group (n = 7), as compared to the responders (n = 3) (p = 0.042) (Figure 2J and 2K, respectively). There were not glands present in all samples why the number of samples analyzed is less for S-1, and only one sample from the NR group had glands, why we could do no statistical comparison to that group. The squamous epithelium was strongly stained in all groups (Figure 2L). A representative image of the negative control for the monoclonal antibodies is shown in Figure 2N.

### Estrogen receptors and Ki-67

#### ERα

**Figure 5:**
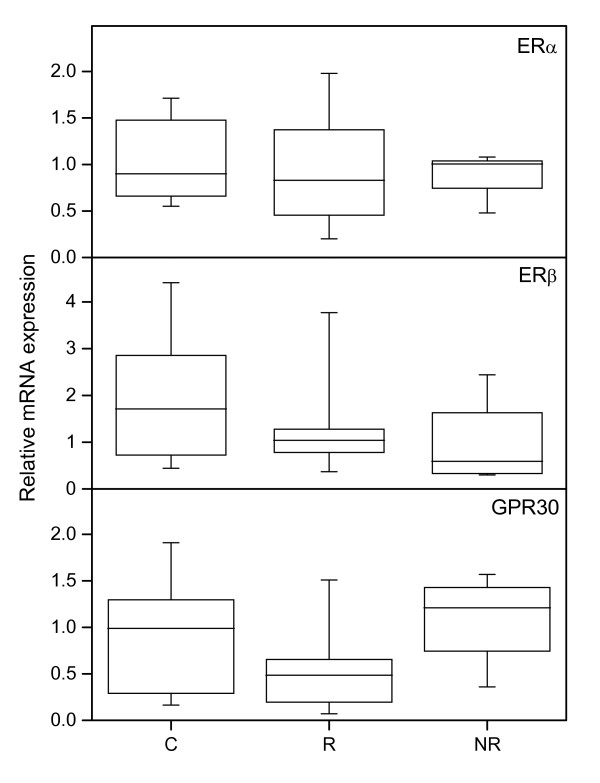
**PCR results for ERα, ERβ and GPR30**. Representative real-time PCR experiments for expression of ERα (upper), ERβ (middle) and GPR30 (bottom) mRNAs in human cervix from the C (n = 11), R (n = 10) and NR (n = 4) groups respectively. The values of relative expression of target genes were normalized against cyclophilin A and displayed in arbitrary units. The "box-and-whisker plot" represents the median value with 50% of all data falling within the box. The whiskers extend to the 5th and 95th percentiles.

**Figure 6:**
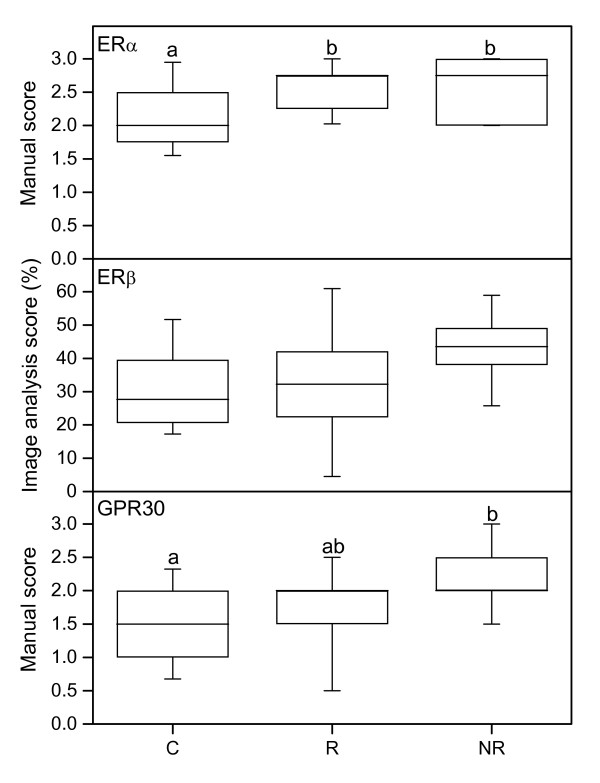
**Immunostaining results of ERα, ERβ and GPR30**. ERα (top), ERβ (middle) and GPR30 (bottom) immunostaining results in stroma, as assessed by manual scoring (top, bottom) and image analysis (middle) in cervical samples from controls (C) (n = 18), responders (R) (n = 13) and non-responders (NR) (n = 9). The "box-and-whisker plot" represents the median value with 50% of all data falling within the box. The whiskers extend to the 5th and 95th percentiles. Boxes with different letter designations are significantly different, p < 0.05.

**Figure 7:**
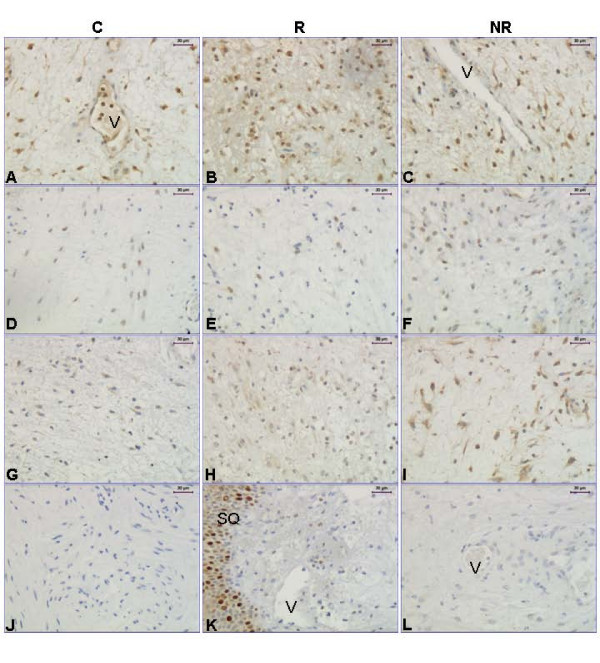
**Representative images of ERα, ERβ, GPR30 and Ki-67 immunostaining**. Representative images of the immunostaining results for ERα (**A-C**), ERβ (**D-F**), GPR30 (**G-I**) and Ki-67 (**K**), and a negative control for monoclonal antibodies (**J**) where the primary antibody (in this example ERα) was replaced by an equal amount of mouse IgG. A negative control for the GPR30 polyclonal antibody, where the primary antibody was replaced by an equal amount of goat IgG, is shown in (**L**). Abbreviations: V: vessel and SQ: squamous epithelium. Magnification bars represent 30 μm in all images.

There are no significant differences in the mRNA level between the study groups (Figure 5, upper panel), but the protein level in stroma was significantly lower in the control group as compared to the R and NR groups (Figures 6, upper panel; Figure 7A–C).

#### ERβ

No significant differences in the mRNA level between groups were found (Figure 5, middle panel). A tendency to more immunostaining in the stroma was found in the NR group as compared to controls (p = 0.073) (Figure 6, middle panel; Figure 7D–F).

#### GPR30

The mRNA levels of GPR30 did not differ between groups (Figure 5, bottom panel). Immunostaining of GPR30 in cervical stroma was stronger in the NR group as compared to controls (Figure 6, bottom panel; Figure 7G–I).

#### Ki-67

Immunostaining with the antibody directed towards the proliferation marker Ki-67 showed no differences between groups (data not shown). Typically the squamous epithelium was positive while most stroma cells were negative (Figure 7K).

A representative image of the negative controls for the monoclonal antibodies is shown in Figure 7J, while a negative control for the polyclonal GPR-30 antibody is shown in Fig 7L.

## Discussion

Impaired cervical ripening is a dominating feature in post term pregnancy. The trigger initiating the normal cervical softening including remodelling of the cervical ECM is unclear. The 100-fold increase of estradiol and progesterone concentrations, as a result of the placental production, in peripheral maternal blood at term pregnancy may support a role for hormonal regulation. Furthermore, the significant higher level of fetal fibronectin in cervical mucus after successful ripening with prostaglandin-E2 supports a possible fetal role in the onset of labour [31].

In previous studies we have been able to show that there are decreased levels of stromal ERα and ERβ in human cervix immediately post partum as compared to the non-pregnant state and term, respectively [13]. In the present study the NR group showed an increased level of ERα immunostaining compared to the controls whereas ERβ showed a tendency to increased immunostaining, indicating a possibility of increased ERα and ERβ levels to be a sign of decreased spontaneous cervical ripening. However, there were no differences in Ki-67 immunostaining between groups, indicating no change in estrogen response regarding proliferation in the cervix.

In studies on human leukocytes we have found expression of both ERα and ERβ, the mRNA in several splice variants and the protein in different sizes both in mononuclear and polymorphonuclear cells [32]. Leukocytes from rats treated with selective ER agonists expressed several genes associated to extracellular matrix remodelling [33]. Further studies to evaluate if these genes are involved in the cervix remodelling in humans are needed.

The recent discovery of GPR30 as an estrogen binding membrane receptor, made us interested to describe its expression in the human cervix. GPR30 immunostaining was significantly increased in the NR group as compared to controls. Thus, this estrogen binding protein behaved similarly to ERα and ERβ. More studies are needed to elucidate this increase in estrogen binding proteins in the cervix from post term non-responders compared to those that delivered spontaneously at term. In the hamster ovary GPR30 was shown to be regulated by the gonadotropins LH and FSH, whereas estrogen and progesterone had no effect, when given to hypophysectomized animals [34].

Cervical ripening has been shown to involve an inflammatory reaction, including leukocyte influx and activated fibroblasts [3-6,20,22,35,36]. In the present study it is notable that IL-8, PAF-R and MMP-9, all predominantly expressed in leukocytes, are significantly reduced in the NR group. For IL-8, the level in the NR group is comparable to that in cervix from non-pregnant women. The decrease found could be a secondary effect due to the impaired accumulation of leukocytes in the non-responders, since they demonstrated a lower level of leukocytes as measured by CD45 immunoreactivity, in comparison to the group responding to prostaglandin treatment.

We have previously shown that CD45 positive cells are increased in the cervix of women in term pregnancy and immediately post partum as compared to the non-pregnant [3]. No differences were found between term and post partum, hence the actual delivery does not seem to change the leukocyte influx. This differs from the results by Timmons and Mahendroo, who showed that in mice the leukocyte invasion occurs in labour and not due to ripening of the cervix. They also conclude that a role for neutrophils is more probable in postpartum remodelling of the cervix, rather than in the initiation of cervical ripening at parturition [37]. Another group stated though that the above study is the only one showing no increase of neutrophils prior to parturition in mice, and speculate that the different way of measuring immune cells (in a suspension of tissue with a hemacytometer) could be the reason for the different result [38]. Yellon et al. concluded that wild-type mice demonstrate an increased presence of macrophages, as well as reduced collagen structure in the cervix in late pregnancy, which is consistent with the hypothesis that recruitment of macrophages is important for cervical remodelling before term [38].

In the present study the influx of CD45 positive leukocytes is strongest in the R group, thereafter the C group and significantly lower in the NR group. Thus, if the post-term women were able to react on prostaglandin treatment the accumulation of leukocytes was restored to, or even tended to overcome, that of the level in the cervix of women delivering spontaneously at term. Post term women could be divided into at least two subgroups, those with non-ripe cervices that with the correct stimulus, like the prostaglandin gel, could progress into labour and a vaginal delivery; and those that fail to respond to prostaglandin stimulation and have to be delivered by caesarean section.

In term and preterm cervical ripening mRNAs of the three isomers of NOS, nNOS, eNOS and iNOS have been identified. An increased expression of nNOS was registered at preterm cervical ripening [39]. One of the few articles which present results from studies on cervix in post term women showed reduced cervical nitric oxide release in cervical fluid of post term compared to term women [40]. This decrease in NO levels is in agreement with the decreased level of leukocytes found in our study, since certain leukocytes are known to express iNOS [41].

Platelet activating factor (PAF) is a pro-inflammatory mediator, which has been implicated in parturition. Local application of PAF in rats induced cervical ripening [35], whereas a PAF-receptor (PAF-R) antagonist prolonged parturition [42]. The PAF-R has been identified in human cervical fibroblasts in vitro [43]. We have previously shown presence of the PAF-R protein in the human uterine cervix *in vivo *[20]. Stromal PAF-R immunostaining was most pronounced at term, and decreased after parturition. PAF increases the expression of pro-inflammatory cytokines e.g. IL-8, and this effect can be abolished using a PAF-R antagonist (WEB2170) [17,43]. In the immortalized sebaceous gland cell line SZ95 PAF-R activation has been shown to induce expression of cyclooxygenase-2, prostaglandin-E2 and IL-8 [44]. Thus, the PAF-R has the potential to induce these factors, all which have been shown to be involved in the process of cervical ripening [4,20]. In the present study the PAF-R expression was lower in the NR group, possibly due to the lower level of leukocytes present in this group. The amount of PAF-R might require a certain level to be able to induce cervical ripening in response to activation. Lack of activation might then reduce or inhibit the pro-inflammatory modulators that have been shown to be the response to PAF-R activation [21]. This is well in agreement with our present results showing a lower level of IL-8 in the post term group not responding to prostaglandin priming.

The remodelling of the cervical ECM includes both increased synthesis as well as degradation of collagen fibrils and proteoglycans. MMP-2 and MMP-9 expression in human cervix increase at term pregnancy, suggesting involvement of these proteases in the reorganization of ECM during cervical ripening. In the human cervix, MMP-2 and MMP-9 are produced by different cell types: MMP-2 was immunolocalized mainly to stromal fibroblasts and smooth muscle cells, whereas MMP-9 was observed exclusively in leukocytes [22]. Taken together with our present results, data indicate the importance of leukocyte derived MMP-9 for a spontaneous cervical ripening process. Leukocytes are recognized as a main source of the catabolic enzymes involved in the remodelling of the cervix during parturition [45]. The present results suggest that the lower level of leukocytes lead to a less intensive inflammatory event and therefore impaired or absent cervical ripening. The decrease in MMP-9 and PAF-R could be secondary to the lower leukocyte count.

The syndecan family of transmembrane proteoglycans is the major source of cell surface heparane sulphates (HS) on all cell types. Recent *in vitro *and *in vivo *data suggest the involvement of syndecans in the modulation of leukocyte-endothelial interactions and extravasation, the formation of chemokine gradients, participation in chemokine and growth factor signalling, as well as repair processes [46]. In vitro IL-8 has been shown to shed from endothelial cells bound to S-1 and HS, and increased shedding was accompanied by a significant decrease in the number of neutrophils migrating across the endothelium [47]. Thus, S-1 could be involved in the influx of leukocytes and the cytokine presentation in the tissue. We found an increased level of S-1 adjacent to glands in the controls, where we also found strong immunostaining for IL-8. In two recent articles S-1 expression in human endometrium during the menstrual cycle has been described [48,49]. Although the function of S-1 in normal endometrium is not known, the changes in expression related to the hormonally regulated menstrual cycle [48,49] and after estrogen treatment to ovariectomized rats [26], suggest a possible role of S-1 in hormone regulated tissue remodelling. In addition S-1, as well as Ki-67, is primarily expressed in cervix epithelium, where also ERα and ERβ expression is most pronounced [13].

## Conclusion

Failed induction of cervical ripening and delivery of post term pregnant women was associated with decreased accumulation of leukocytes, possibly resulting in the lower levels of factors important for remodelling of the ECM, such as PAF-R and MMPs determined in the present study. If the decreased leukocyte influx is the primary cause, or the secondary result of another factor, maternal and/or fetal, have to be investigated further.

## Competing interests

The authors declare that they have no competing interests.

## Authors' contributions

LS, YSV and GEO participated in the design of the study. All samples were collected by YSV. The analyses of proteins and mRNAs were carried out by BM and LS. Data analyses were performed by BM and LS. NR and GEO have helped to draft the manuscript, and the manuscript was written by LS. All authors have read and approved the final manuscript.
